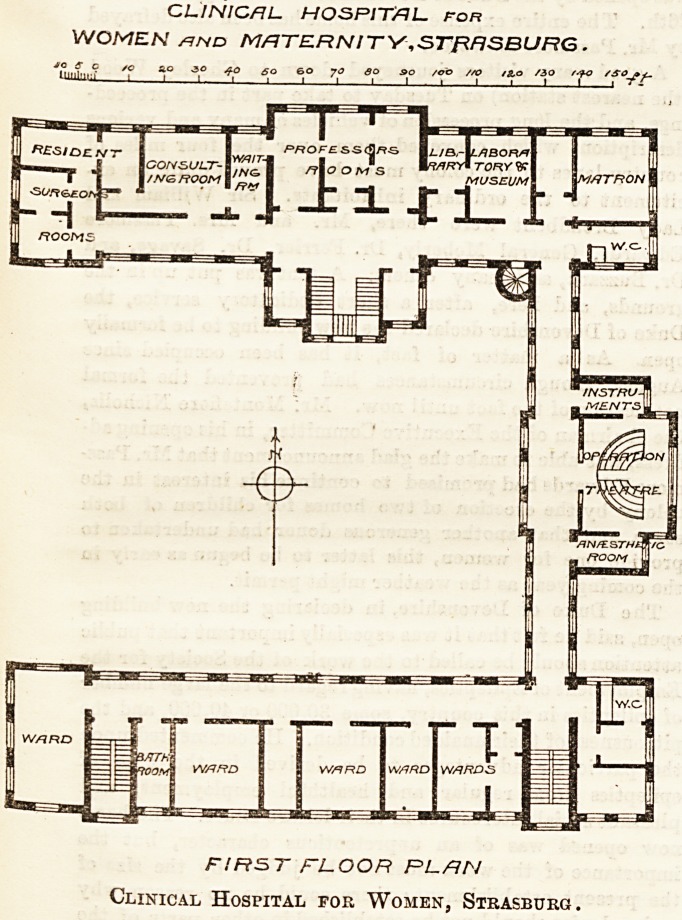# Hospital Construction

**Published:** 1895-12-07

**Authors:** Charles Bodon

**Affiliations:** of Budapest.


### The Institutional Workshop.

### HOSPITAL CONSTRUCTION.

### STRASBURG HOSPITAL FOR WOMEN.

By Charles Bodon, M.D., of Budapest.

This institution serves the double purpose of a hospital for women and a lying-in hospital. As it is considered to be the best, or at least one of the best hospitals of this kind in Germany, it may be worth while to give a short description of it.

The fine building was opened in 1886. It is situated far from the centre of the town, but yet within the fortifications. Its shape is that of an

FIRST FLOOR PLAN

Clinical Hospital for Women, Strasburg.

angular horse-shoe, being open to the west, because it is this way that the summer winds generally blow.

The building is two storeys high. On the ground floor are the students' rooms, a large dining-room, and four dormitories for four to six pregnant women each, besides attendants' rooms and administrative offices.

On the first floor, as shown in the plan, is a very fine operating theatre. Its lighting is effected by a large window as well as by a top light. It is floored with terrazzo. The walls can be sluiced down with disinfectants. The theatre is adjoined on one side by the anæsthetic room, on the other by the sterilising room for instruments and dressings. The wards on this floor give accommodation for patients requiring gynæcological treatment. Each ward contains five to eight beds. There is besides a consulting and a waiting room for out-patients.

The second floor serves as a lying-in hospital. There are two separate rooms for women in labour, and five wards for puerperæ. In the garden of the hospital there is a small isolation building, with two wards for patients suffering from infectious diseases.

The average number of gynæcological cases treated amounts to 450, and of confinements to 400 a year. The gynæcological service is carried out by three resident surgeons, three Sisters, and seven nurses; the obstetrical service by one resident physician, two midwives, and three nurses.

As I have had the rare opportunity to see many hospitals in Great Britain, France, Austria-Hungary, Belgium, Italy, Switzerland, and Germany, I might, perhaps, be allowed to utter my opinion about the hospital in question.

Although I do not think it to be quite right that one floor should serve as a gynæcological, and another floor of the very same building as a lying-in hospital, I am still glad to join the general opinion that it has been erected according to the newest requirements of hygiene, and everything fitted and finished so as to meet septic principles. And yet in spite of cleanliness and plenty of air and light, this hospital—like most hospitals I have seen on the Continent—is lacking that particular feeling of homeliness which is so prevalent in British hospitals, where everything and everybody is bright, cheerful, and contented. I cannot emphasise enough the vast superiority of the hospital institutions of the British Isles to anything I have witnessed on the Continent, and I am bound to think that this difference is due partly to the practical sense and strong charity-feeling of the Anglo-Saxon race, and chiefly to its nurses, who know how to arrange things nicely, how to care for their patients, and how to make them comfortable. Of course there are good nurses on the Continent as well; but, generally speaking, our nurses are neither so intelligent nor so devoted to their work and duty as English nurses are.

## Figures and Tables

**Figure f1:**